# Evidence of indirect gap in monolayer WSe_2_

**DOI:** 10.1038/s41467-017-01012-6

**Published:** 2017-10-13

**Authors:** Wei-Ting Hsu, Li-Syuan Lu, Dean Wang, Jing-Kai Huang, Ming-Yang Li, Tay-Rong Chang, Yi-Chia Chou, Zhen-Yu Juang, Horng-Tay Jeng, Lain-Jong Li, Wen-Hao Chang

**Affiliations:** 1 Department of Electrophysics, National Chiao Tung University, Hsinchu, 30010 Taiwan; 2 Physical Sciences and Engineering, King Abdullah University of Science and Technology, Thuwal, 23955-6900 Saudi Arabia; 3 Research Center for Applied Sciences, Academia Sinica, Taipei, 10617 Taiwan; 4 Department of Physics, National Cheng Kung University, Tainan, 70101 Taiwan; 5 Department of Physics, National Tsing Hua University, Hsinchu, 30013 Taiwan

## Abstract

Monolayer transition metal dichalcogenides, such as MoS_2_ and WSe_2_, have been known as direct gap semiconductors and emerged as new optically active materials for novel device applications. Here we reexamine their direct gap properties by investigating the strain effects on the photoluminescence of monolayer MoS_2_ and WSe_2_. Instead of applying stress, we investigate the strain effects by imaging the direct exciton populations in monolayer WSe_2_–MoS_2_ and MoSe_2_–WSe_2_ lateral heterojunctions with inherent strain inhomogeneity. We find that unstrained monolayer WSe_2_ is actually an indirect gap material, as manifested in the observed photoluminescence intensity–energy correlation, from which the difference between the direct and indirect optical gaps can be extracted by analyzing the exciton thermal populations. Our findings combined with the estimated exciton binding energy further indicate that monolayer WSe_2_ exhibits an indirect quasiparticle gap, which has to be reconsidered in further studies for its fundamental properties and device applications.

## Abstract (web summary)

Monolayer transition metal dichalcogenides have so far been thought to be direct bandgap semiconductors. Here, the authors revisit this assumption and find that unstrained monolayer WSe_2_ is an indirect-gap material, as evidenced by the observed photoluminescence intensity-energy correlation.

## Introduction

Two-dimensional (2D) transition metal dichalcogenides (TMDs) in the family of MX_2_ (M:Mo, W; X:S, Se) have been discovered as a new class of semiconductors for atomically thin electronics and optoelectronics^1–6^. Of particular interest are monolayer MX_2_, which have been identified as direct gap semiconductors and emerged as new optically active materials for developing novel 2D light emitters and absorbers^5, 6^. Experimental evidence of direct gap in monolayer MX_2_ was obtained from the observation of strongly enhanced photoluminescence (PL) caused by the indirect to direct gap transition when MX_2_ were thinned to one monolayer^7, 8^. Although most theoretical calculations have also shown a direct gap at K-valley in all monolayer MX_2_^9–12^, subtle differences have been found in some calculations for monolayer WSe_2_^13^, where the conduction band (CB) minimum lies at Q-valley (about midway between K and Γ). Indeed, recent scanning tunneling spectroscopy (STS) suggested that the Q-valley is about 80 meV below the K-valley in the CB of monolayer WSe_2_^14^, indicative of an indirect quasiparticle gap. However, due to the unusually strong Coulomb interactions and the lack of knowledge on the binding energy of indirect excitons, it remains unclear whether the optical gap (i.e., quasiparticle gap minus exciton binding energy) is also indirect. While efficient PL is invariably observed in monolayer WSe_2_, the indirect quasiparticle gap observed by STS suggested that the direct/indirect nature of optical gap in monolayer WSe_2_ has not been settled thus far.

Strain is an effective perturbation that can modulate not only the direct gap of MX_2_, but also the indirect gap with a different trend^11, 15–18^. As the indirect gap energy is pushed away from or brought closer to the direct gap by strain, the exciton population participating in the direct-gap recombination will be affected by the presence of nearly degenerated indirect gap, leading to a modulation in the energy and intensity of direct exciton PL.

In this work, instead of applying external stress, we investigate the spatially resolved PL from monolayer WSe_2_–MoS_2_ and MoSe_2_–WSe_2_ lateral heterojunctions (HJs) with inherent nonuniform strain distribution^19^. The built-in local strain inhomogeneity exhibited in lateral HJs provides a unique platform to study the strain effects on MX_2_ without the need of applying strain. Here we first demonstrate PL from strained MoS_2_ in WSe_2_–MoS_2_ lateral HJs as a model system to extract the energy difference between the direct and indirect gaps in monolayer MoS_2_. Then we demonstrate the study of MoSe_2_–WSe_2_ lateral HJs and show that unstrained monolayer WSe_2_ is actually an indirect gap material.

## Results

### PL inhomogeneity in strained WSe_2_–MoS_2_ lateral HJs

**Fig. 1:**
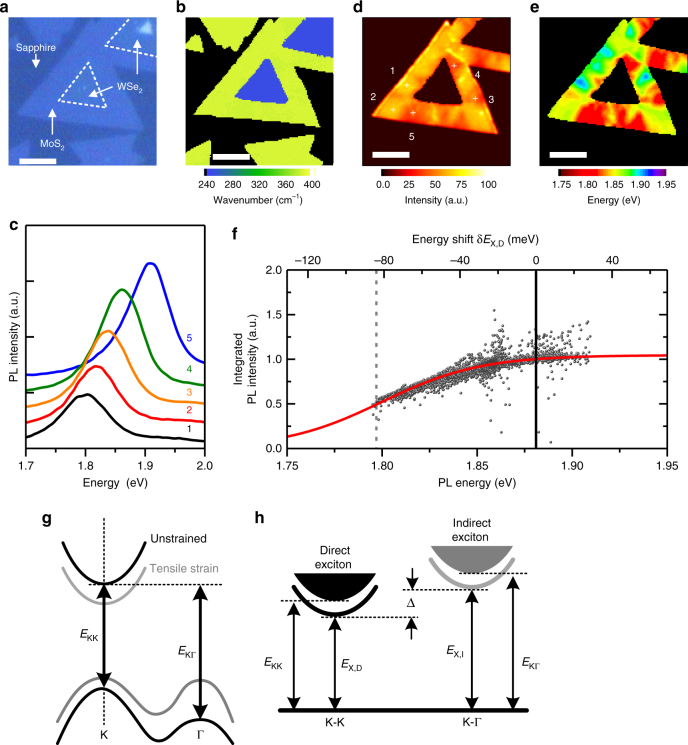
PL inhomogeneity in strained WSe_2_–MoS_2_ lateral HJs. **a**, **b** Optical image (**a**) and the corresponding contour color map of the E_2g_ Raman peak position (**b**) of a monolayer WSe_2_–MoS_2_ lateral HJ flake. **c** Five selected PL spectra from different positions in the MoS_2_ region. **d**, **e** Contour color maps of the PL intensity (**d**) and peak energy (**e**) of the MoS_2_ region. **f** Integrated PL intensity as a function of peak energy obtained from all PL spectra (black dots). Solid line is the model fitting curve. The top axis is the PL peak energy shift with respect to the unstrained MoS_2_ (vertical line). **g** Schematics for the band structures of monolayer MoS_2_ without (black) and with (gray) tensile strain. **h** Schematics for the direct (K-K) and indirect (K-Γ) excitonic states with energies of *E*_X,D_ and *E*_X,I_, respectively

Figure 1a shows the optical image of a typical WSe_2_–MoS_2_ lateral HJs. Raman analysis (Fig. 1b) reveals that the inner and outer regions are monolayer WSe_2_ and MoS_2_, respectively. The typical size of the HJ flakes is ~ 15–25 μm. According to ref. ^19^, the outer MoS_2_ exhibits nonuniform strain distribution due to the lattice mismatch between two materials, leading to considerable spatial variations in local PL spectra in the outer MoS_2_. Figure 1c shows five selected PL spectra, of which the PL energy variation is ~ 100 meV, corresponding to a strain variation up to ~ 2% in the monolayer MoS_2_ (based on the reported linear PL energy shift rate of ~ 45 meV/% strain)^16^. As shown in Fig. 1d and e, the spatial distributions of PL intensity and energy in outer MoS_2_ show a “positive” correlation, i.e., the site with a higher PL energy always exhibited a higher intensity. The PL intensity–energy correlation can be seen clearly in Fig. 1f, where the integrated intensities and peak energies collected from all local PL spectra of the outer MoS_2_ are plotted together.

The PL intensity–energy correlation arises from the variation of direct exciton populations caused by the strain-induced changes in the direct and indirect gap energies. For monolayer MoS_2_, the indirect gap (K-Γ) is higher in energy than the direct gap at K-valley (Fig. 1g)^20–22^. Since the K-Γ indirect gap (*E*_KΓ_) reduces with tensile strain faster than the K-K direct gap (*E*_KK_)^11, 15–17^, the direct and indirect exciton states will be brought closer in energy by tensile strain, leading to a marked change in direct exciton population and hence the PL intensity. We analyze the steady-state PL intensity by modeling the exciton populations in a simple two-level system (Supplementary Fig. 1 and Supplementary Note 1). In thermal equilibrium, the populations of direct *N*_X,D_ and indirect *N*_X,I_ excitons follow the Boltzmann distribution *N*_X,I_ = *N*_X,D_exp(−Δ/*k*_B_ *T*), and

1 $${N_{X,D}} = \frac{{{N_0}}}{{{\rm{exp}}( - \Delta {\rm{/}}{k_{\rm B}}T) + 1}},$$

where Δ = *E*_X,I_−*E*_X,D_ is the energy difference between the indirect (*E*_X,I_) and direct (*E*_X,D_) exciton energies (Fig. 1h), and *N*_0_ = *N*_X,D_ + *N*_X,I_ is the total exciton population. The energy difference Δ is strain dependent, which can be expressed as $\Delta = ( {E_{{\rm{X}},{\rm{I}}}^0 + {\rm{\delta }}{E_{{\rm{X}},{\rm{I}}}}} ) - ( {E_{{\rm{X}},{\rm{D}}}^0 + {\rm{\delta }}{E_{{\rm{X}},{\rm{D}}}}} ) = {\Delta _0} + (\gamma - 1)\delta {E_{{\rm{X}},{\rm{D}}}}$, where $E_{{\rm{X}},{\rm{D}}}^0$ and $E_{{\rm{X}},{\rm{I}}}^0$ (δ*E*_X,D_ and δ*E*_X,I_) are the unstrained (strain-induced changes in) energies of the direct and indirect excitons, respectively, ${\Delta _0} = E_{{\rm{X}},{\rm{I}}}^0 - E_{{\rm{X}},{\rm{D}}}^0$ is the unstrained value of Δ, and *γ* = δ*E*_X,I_/δ*E*_X,D_, which is related to the ratio of deformation potential of the K-Γ and K-K quasiparticle gaps. The strain-induced shift in the direct exciton energy, i.e., ${\rm{\delta }}{E_{{\rm{X}},{\rm{D}}}} = {E_{{\rm{X}},{\rm{D}}}} - E_{{\rm{X}},{\rm{D}}}^0$, can be obtained from the difference between PL peak energies of strained and unstrained monolayer MoS_2_. In our analysis, we assumed the PL peak energy of transferred MoS_2_ monolayers on sapphire substrates (1.881 eV; vertical line in Fig. 1f) as the direct exciton energy of unstrained monolayer MoS_2_ (Supplementary Fig. 2). By fitting Eq. (1) to the experimental data and considering Δ_0_ and *γ* as fitting parameters, we obtain a very good fit (solid curve in Fig. 1f) and determine Δ_0_ = 0.08 eV and *γ* = 1.95 ± 0.14 (see Supplementary Figs. 3 and 4), indicative of a direct optical gap in unstrained MoS_2_ monolayer, consistent with past understandings^7, 8, 20–22^.

### PL inhomogeneity in strained MoSe_2_–WSe_2_ lateral HJs

**Fig. 2:**
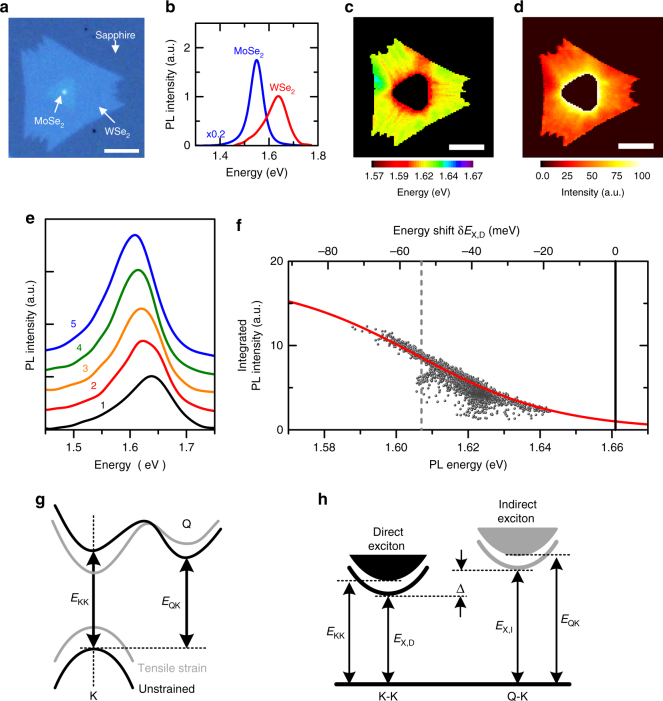
PL inhomogeneity in strained MoSe_2_–WSe_2_ lateral HJs. **a** Optical image of a monolayer MoSe_2_–WSe_2_ HJ. **b** The PL spectra of the inner MoSe_2_ and the outer WSe_2_. **c**, **d** Contour color maps of the PL peak energy (**c**) and intensity (**d**) of outer WSe_2_. **e** Five selected PL spectra from the outer WSe_2_. (**f**) Integrated PL intensity as a function of peak energy (black dots). Solid line is the model fitting curve. The top axis is the PL peak energy shift with respect to the unstrained WSe_2_. **g** Schematics for the band structures of monolayer WSe_2_ without (black) and with (gray) tensile strain. **h** Schematics for the direct (K-K) and indirect (Q-K) excitonic states with energies of *E*_X,D_ and *E*_X,I_, respectively

We now turn to the study of strained WSe_2_. Figure 2a shows the optical image of a typical MoSe_2_–WSe_2_ lateral HJs, where the inner and outer regions have been identified as MoSe_2_ and WSe_2_ monolayers, respectively, by PL measurements (Fig. 2b). As shown in the spatially resolved PL measurements (Fig. 2c, d) and the selected PL spectra from different areas (Fig. 2e), the outer WSe_2_ region also exhibits remarkable variations in local PL intensity and energy due to the spatial strain inhomogeneity. Since the lattice mismatch between MoSe_2_ and WSe_2_ is small (<0.2%)^23^, the strain inhomogeneity is likely to arise from the cooling process after high-temperature growth owing to the mismatch in thermal expansion coefficients between TMDs and substrates^24–27^. In contrary to strained MoS_2_, the PL intensity and energy of the strained WSe_2_ shows a “negative” correlation, i.e., the site with a higher PL energy exhibited a lower PL intensity (Fig. 2f). The negative intensity–energy correlation suggests that the strain-induced change in the indirect gap is very different from that in MoS_2_. In monolayer WSe_2_ (Fig. 2g), the Q-K indirect gap is very close to K-K direct gap. Under tensile strain, the K-K direct gap reduces, while the Q-K indirect gap increases^18^, leading to the increasing PL intensity with tensile strain.

We use the same model to analyze the PL intensity–energy correlation of strained WSe_2_, but now the indirect exciton represent that formed at the Q-K indirect gap (Fig. 2h). We have also determined $E_{{\rm{X}},{\rm{D}}}^0 = 1.661\,{\rm{eV}}$ for unstrained monolayer WSe_2_ (vertical line in Fig. 2f) according to PL measurements on transferred WSe_2_ monolayers (Supplementary Fig. 2). As shown by the solid curve in Fig. 2f, the model fits the data very well, yielding Δ_0_ = −0.07 eV and *γ* = −0.32 ± 0.05 (Supplementary Fig. 5). Interestingly, both Δ_0_ and *γ* for monolayer WSe_2_ are negative, in stark contrast to monolayer MoS_2_. The negative *γ* arises from the opposite energy evolutions of Q and K valleys with strain^18^, while the negative Δ_0_ is a direct evidence of indirect optical gap in unstrained monolayer WSe_2_. The indirect gap of monolayer WSe_2_ also manifested itself in the large variation in PL intensity (up to a factor of ~ 5) shown in Fig. 2f, because, according to Eq. (1), the increase in PL intensity by tensile strain should be less than a factor of two if monolayer WSe_2_ is initially a direct gap material.

### Calculated strain-induced bandgap shifts in MoS_2_ and WSe_2_ monolayers

**Fig. 3:**
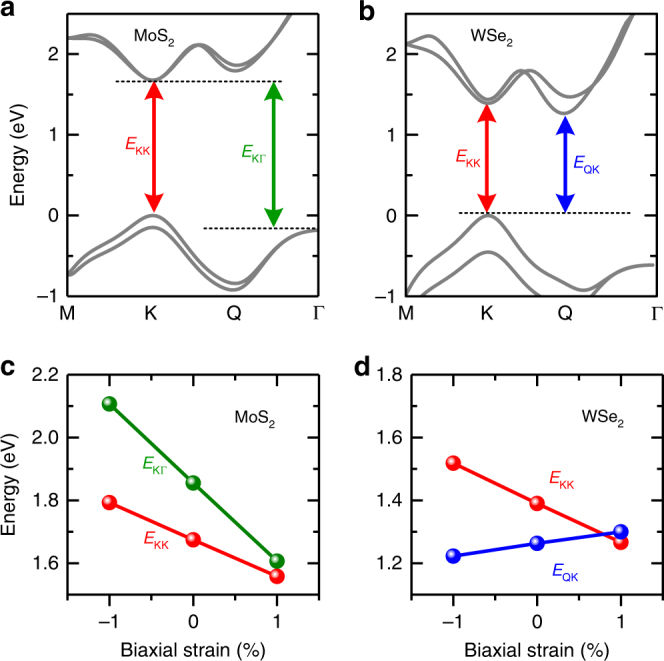
DFT calculations of strained MoS_2_ and WSe_2_ monolayers. **a**, **b** The calculated band structures for monolayer MoS_2_ (**a**) and WSe_2_ (**b**) based on DFT. *E*_KK_ and *E*_KΓ_ (*E*_QK_) denote the direct and the indirect gaps in MoS_2_ (WSe_2_), respectively. **c**, **d** Strain-induced energy shifts in the direct and indirect gaps in monolayer MoS_2_ (**c**) and WSe_2_ (**d**). The calculations show that unstrained WSe_2_ is an indirect gap material, which can be turned into a direct-gap material by applying tensile strain

Band structure calculations based on density functional theory (DFT) have been performed and compared to our experimental results. As shown in Fig. 3a, the calculated band structure for unstrained monolayer MoS_2_ exhibits a direct gap at the K valleys. The calculated energies of the K-K and K-Γ gaps as a function of strain (Fig. 3c) show a faster decrease in *E*_KΓ_ with tensile strain than *E*_KK_. If we ignore the strain-induced change in exciton binding energy, the ratio of strain-induced changes in the K-Γ and K-K gaps (i.e., δ*E*_KΓ_/δ*E*_KK_) thus represents the ratio of strain-induced changes in the optical gaps *γ* = δ*E*_X,I_/δ*E*_X,D_. From the calculations, we determine δ*E*_KΓ_/δ*E*_KK_ = 2.13, consistent with our experimentally obtained *γ* = 1.95 ± 0.14 for monolayer MoS_2_. On the contrary, our calculations show that unstrained monolayer WSe_2_ exhibits an indirect gap between the Q-valley in the CB and the K point in the valence band (VB), as shown in Fig. 3b. Applying tensile strain (Fig. 3d) tends to shrink the K-K gap, but expand the Q-K gap. We determine δ*E*_QK_/δ*E*_KK_ = −0.28, in good agreement with our experimental value of *γ* = −0.32 ± 0.05 for monolayer WSe_2_.

We emphasize that the experimentally determined Δ_0_ does not reflect the actual energy difference between the direct and indirect quasiparticle gaps. Due to the strong exciton binding energy caused by reduced dielectric screening in monolayer TMDs^6, 28, 29^, the difference in binding energies of direct and indirect excitons has to be taken into account. We first calculate the direct exciton binding energy *E*_B,D_ based on the effective 2D Coulomb potential (Supplementary Fig. 6 and Supplementary Note 2). The indirect exciton binding energy *E*_B,I_ is then estimated by scaling with the effective reduced masses deduced from band structure calculations. We obtain *E*_B,D_ = 0.23 eV (K-K) and *E*_B,I_ = 0.26 eV (K-Γ) for monolayer MoS_2_. The higher *E*_B,I_ by 0.03 eV, originating from the heavier hole effective mass at Γ-valley, implies that the experimentally determined Δ_eV_ = 0.08 *eV* underestimates the actual energy difference between the K- and Γ-valleys in the VB, yielding Δ_K−Γ_ = *E*_KΓ_−*E*_KK_≈0.11 eV, in good agreement with the value in the range of 0.13–0.30 eV determined by angle-resolved photoemission spectroscopy^20–22^. For monolayer WSe_2_, due to the similar effective mass of electrons at K- and Q-valleys in the CB, the deduced *E*_B,D_ = 0.19 eV (K-K) and *E*_B,I_ = 0.20 eV (Q-K) are also similar, yielding Δ_K−Q_ = *E*_QK_−*E*_KK_≈−0.06 eV. It demonstrates that monolayer WSe_2_ exhibits not only an indirect optical gap, but also an indirect quasiparticle gap with a CB minimum at the Q-valley, which is ~ 60 meV lower than the K-valley, consistent with STS measurements^14^.

### Temperature dependence of PL intensity–energy correlation

**Fig. 4:**
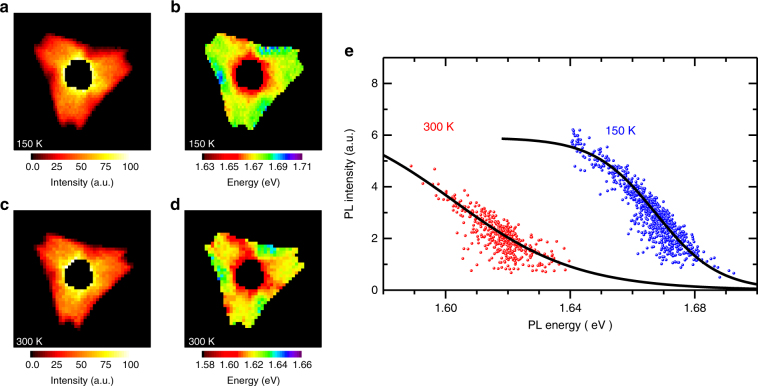
PL intensity–energy correlations at different temperatures. **a**–**d** Contour color maps of PL intensity (**a**, **c**) and peak energy (**b**, **d**) in the WSe_2_ region of a MoSe_2_–WSe_2_ lateral HJ measured at different temperatures: (**a**, **b**) *T* = 150 K and (**c**, **d**) *T* = 300 K. **e** Integrated PL intensity as a function of peak energy measured at 150 K (blue dots) and 300 K (red dots). Black lines are model fitting curves

We noted that the direct exciton populations in the simple two-level model depend sensitively on temperature. To attest the validity of our model analysis, we have performed the same measurements of spatial PL mapping on another MoSe_2_–WSe_2_ HJ flake at T = 150 and 300 K, as shown in Fig. 4a–d. The corresponding PL intensity–energy correlations of the outer monolayer WSe_2_ at 150 and 300 K are shown in Fig. 4e. Apart from a rigid shift in PL energy, the PL intensity measured at T = 150 K exhibits a sharper change with energy due to the reduced thermal energy *k*_B_ *T* at a lower temperature. The simple two-level model can fit the two data sets with the same Δ_0_ and *γ*-values (solid curves in Fig. 4e) by simply changing the sample temperature and the unstrained PL peak energy. The temperature dependent measurements thus further confirm that the observed PL intensity–energy correlation is governed by the interplay of direct and indirect exciton populations. The nonuniform distribution of nonradiative defects or inhomogeneous alloy mixing can thus be excluded from the origin of the observed PL intensity–energy correlation.

## Discussion

In our analysis, we neglect the influence of dark exciton states at K valleys arising from the spin splitting in the CB. In particular, the spin-forbidden K-K dark exciton state is lower in energy by ~ 30 meV in monolayer WSe_2_^30^, which also affects the bright (direct) exciton populations and hence the PL intensity at different temperatures. However, because the strain-induced change in the CB spin splitting is very small (<0.5 meV/%)^31^, including the dark exciton states will not change the analysis of Δ_0_ and *γ*-values from the PL intensity–energy correlation measured at a given temperature.

Strain could also impact the radiative lifetime and the binding energy of the K-K direct exciton through modifications in wave functions, optical matrix element and band curvatures. However, according to the range of PL shifts in our samples and recent theoretical calculations^32^, we estimated that the strain-induced changes in the radiative lifetime and the binding energy are small (<5%), which is within our experimental uncertainty.

Spatial variation of the unintentional doping in the sample could also lead to PL shifts due to the charged exciton (trion) emissions. We have performed line-shape analysis for the selected PL spectra shown in Fig. 2e and found that no spectral signature can be attributed to the trion emissions (Supplementary Fig. 7). Therefore, spatial variation in doping level can be excluded from the observed PL shift and the intensity–energy correlation.

Alloy inhomogeneity caused by material intermixing could be another source of the observed PL inhomogeneity. We have measured the PL inhomogeneity for HJ flakes after ultrasonic treatments. Interestingly, we found that the spatial distributions of PL intensity and energy are changed, but still exhibit a similar negative correlation (Supplementary Fig. 8). Since the strain inhomogeneity is likely to form during the fast-cooling process caused by the contraction mismatch, the local interactions between TMD and the substrate could be loosen and redistributed by ultrasonic treatments, and thereby changing the pattern of strain inhomogeneity. This result thus excludes the possibility that the PL inhomogeneity is caused by local variation of doping, defect distribution or alloy composition, since they are unlikely to be altered by ultrasonic treatments.

The indirect optical gap in unstrained monolayer WSe_2_ identified in this work has important implications. As a light emitter, monolayer WSe_2_ is far from ideal as compared with other MX_2_ with a direct gap, since a majority of photogenerated carriers will form indirect excitons even at room temperature, which considerably limits the quantum yield of light emissions. On the other hand, the efficient light absorption through the direct optical gap, together with the suppressed radiative recombination efficiency for the indirect excitons, makes monolayer WSe_2_ very ideal for photovoltaic applications. The indirect Q-K exciton in WSe_2_ is essentially dark and inaccessible optically. Recent theoretical work predicted that non-covalently attached molecules with a strong dipole moment can turn the indirect dark exciton bright, implicating its applications in molecule sensing^33^. Nevertheless, applying a moderate tensile strain (~ 1% uniaxial or ~ 0.5% biaxial) can tune the bandgap from indirect to direct, which can improve drastically the emission efficiency by nearly an order of magnitude. Based on the simple two-level model, we estimated that the crossover of the direct and indirect optical gaps occurs at 1.607 eV (1.797 eV) for monolayer WSe_2_ (MoS_2_), as indicated by the vertical dash line in Fig. 2f (Fig. 1f). For the strained MoSe_2_–WSe_2_ lateral HJs investigated here, the PL peak energy of the WSe_2_ region spans from 1.58 to 1.64 eV, which covers the crossover energy of direct and direct optical gap at 1.607 eV. This means that local strain variation also gives rise to a spatial modulation of direct and indirect gap, which forms a very unique platform with nonuniform potential modulations for studying exciton diffusions and localizations. Finally, we point out that the indirect gap nature of monolayer WSe_2_ must to be taken into account in further studies of its fundamental properties (e.g., exciton spin valley dynamics) and device applications for light emissions and harvesting.

## Methods

### Growth of monolayer lateral heterojunctions

High-quality single-crystal TMD HJs were synthesized on sapphire substrates by chemical vapor deposition (CVD) in horizontal hot-wall chambers. The monolayer WSe_2_–MoS_2_ lateral HJs were grown using the two-step growth method (see ref. ^19^ for details), while MoSe_2_–WSe_2_ lateral HJs were grown using the conventional one-pot synthesis process. For WSe_2_–MoS_2_ HJs, we used high-purity WO_3_, MoO_3_, Se and S powders as the source precursors. The growth temperatures for WSe_2_ and MoS_2_ are 925 and 755°C, respectively. For MoSe_2_–WSe_2_ HJs, high-purity MoO_2_, WO_3_, and Se powder were used as the initial reactants, and the HJs were grown at 880°C. All growths were performed in Ar/H_2_ flowing gas at low pressure (5–40 Torr).

### Optical measurements

PL and Raman measurements were performed using a home-built optical microscope in the back-scattering configuration. A 532 nm solid-state laser was used as the excitation source. The laser were focused on the sample by a 100 × objective lens (NA = 0.9). The PL and Raman signals were collected by the same objective, analyzed by a 0.75-m monochromator and detected by a nitrogen-cooled CCD camera. For confocal measurements, the sample image was projected onto a 50 μm pinhole before entering the monochromator, yielding an overall spatial resolution of ~ 0.5 μm. Room temperature spatial mappings of PL spectra were performed using a fast motorized *x–y* stage with a step size of 0.25 μm. Low-temperature measurements were performed in a cryogen-free and vibration-free cryostat equipped with a 3-axis piezo-positioner, an *x-y* scanner and an objective lens (N.A. = 0.82) in the low-temperature chamber.

### DFT calculations

The first-principle calculations were based on the generalized gradient approximation (GGA)^34^ using the full-potential projected augmented wave method^35, 36^ as implemented in the Vienna ab initio simulation (VASP) package^37, 38^. The electronic structures of monolayer MoS_2_ and WSe_2_ were calculated using a 30 × 30 × 1 Monkhorst-Pack *k*-mesh over the Brillouin zone with a cutoff energy of 500 eV under geometry optimization. The spin-orbit coupling was included in a self-consistent manner. Slabs with vacuum thickness larger than 30 Å were used to model the thin films. For the strain effects, the atomic positions were relaxed until the residual forces were less than 0.001 eV/Å.

### Data availability

The data that support the findings of this study are available from the corresponding author upon reasonable request.

## Electronic supplementary material

Supplementary Information
