# Supplementary material for: Evidence of indirect gap in monolayer WSe2
Source: Nat Commun. 2017 Oct 13;8:929. doi: 10.1038/s41467-017-01012-6 (PMC5640683; doi:10.1038/s41467-017-01012-6)
Supplement: Supplementary file 1 — Supplementary Information [file 41467_2017_1012_MOESM1_ESM.pdf]

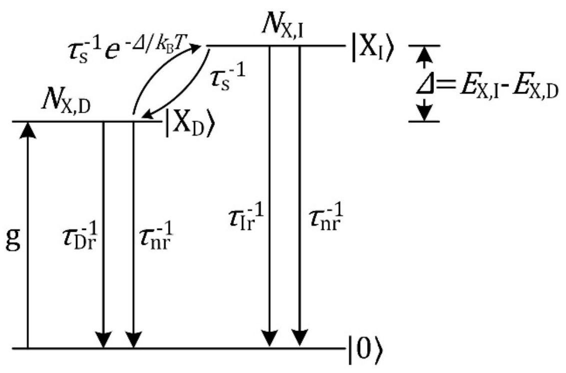

Supplementary Figure 1| Rate-equation model. The populations of direct (indirect) excitons $N_{X,D}$ ($N_{X,I}$) are determined by the exciton generation rate g, the direct (indirect) exciton lifetime τ_D (τ_I) and the exciton intervalley scattering time τ_s, where $\tau_D^{-1} = \tau_{Dr}^{-1} + \tau_{nr}^{-1}$ ($\tau_I^{-1} = \tau_{Ir}^{-1} + \tau_{nr}^{-1}$) includes both the radiative lifetime τ_{Dr} (τ_{Ir}) and non-radiative lifetime τ_{nr} for the direct (indirect) excitons.

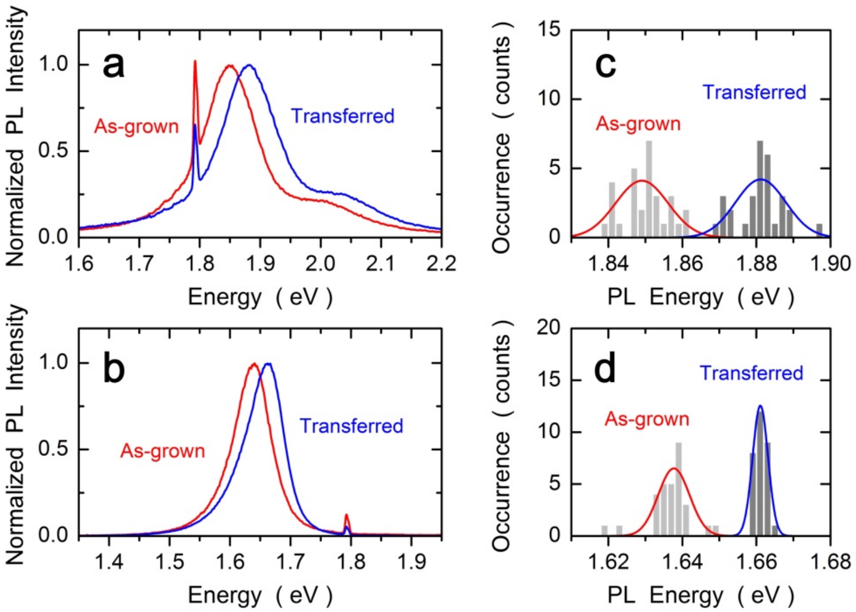

Supplementary Figure 2| PL of as-grown and transferred MoS_2 and WSe_2 monolayer flakes on sapphire substrates. (a,b) PL spectra of CVD-grown monolayer MoS_2 (a) and WSe_2 (b) before and after transfer. The sharp line near 1.8 eV is the emissions from the sapphire substrate. (c,d) Histograms of PL peak energies obtained from 30 monolayer flakes of MoS_2 (c) and WSe_2 (d). CVD-grown samples usually exhibit residual strain induced by cooling process after high-temperature growth due to the large mismatch in thermal expansion coefficients between TMDs and substrates (sapphire or SiO_2/Si), giving rise to $\sim$1% tensile strain in the TMDs [1-4]. The residual strain can be released by transferring the as-grown samples to another clean sapphire substrate. The blueshift of PL peak after transfer indicates that as-grown samples are tensile-strained by the substrate. The transferred samples are assumed to be unstrained. We analyze the peak energy distributions by a Gaussian function. From statistics of 30 monolayer flakes, we determine the PL peak energy of unstrained monolayer MoS_2 and WSe_2 as 1.881 ± 0.006 eV and 1.661 ± 0.002 eV, respectively.

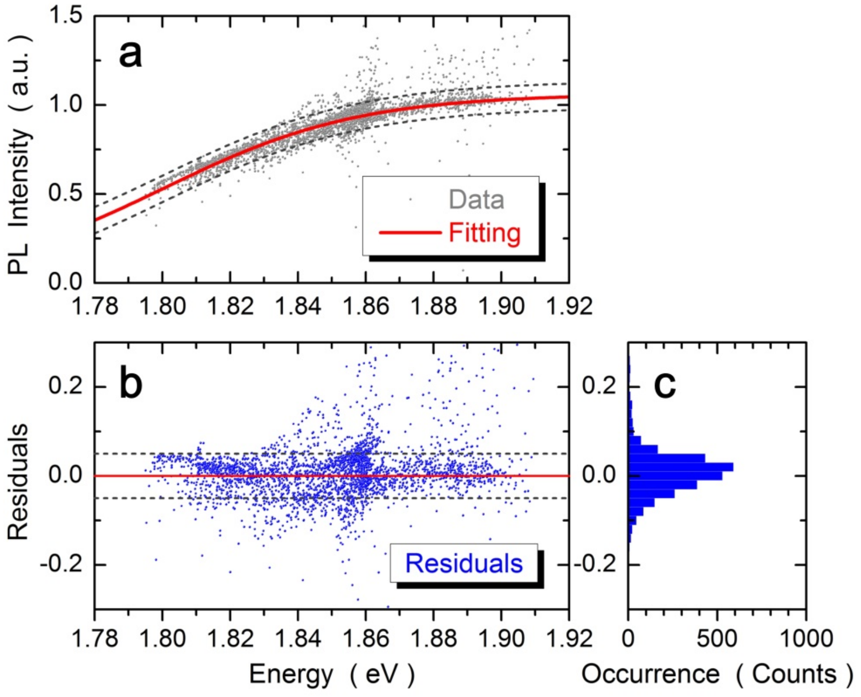

Supplementary Figure 3| Analysis of fitting residuals. (a) Integrated PL intensity as a function of peak energy of monolayer MoS_2. Gray dots: PL data; Red line: The best fitting curve. (b) Residuals of the model fitting. (c) Histogram of the fitting residuals. Dash lines in (a) and (b) indicate the interval of one standard deviation ($\pm\sigma$).

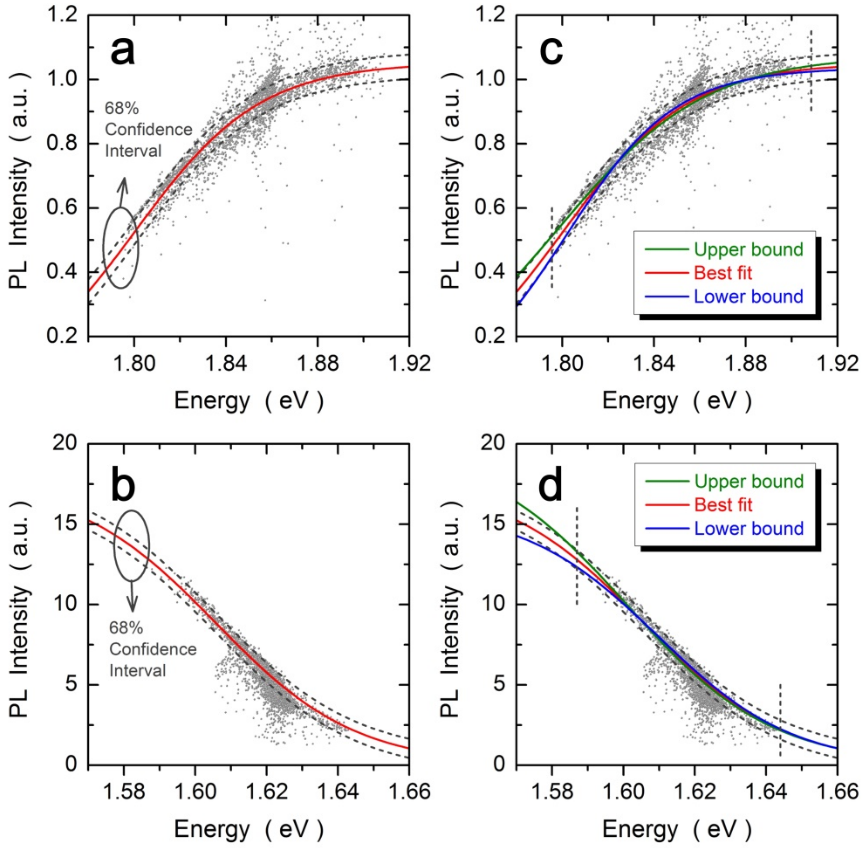

Supplementary Figure 4| Determination of fitting error bars. (a,b) Integrated PL intensity as a function of energy for monolayer MoS_2 (a) and WSe_2 (b). Gray dots: PL data. Red line: the best fitting curve. Dash lines: the interval of one standard deviation ($\pm\sigma$). (c,d) Determination of the upper and the lower bounds of the fitting parameters (Δ_0 and γ) for monolayer MoS_2 (c) and WSe_2 (d). We first determine the interval of $\pm\sigma$ from the best fitting curve, and then change the fitting parameters (Δ_0 and γ) such that the fitting curve remains in the interval within the energy range of experimental data. The error bars were finally determined by the upper and the lower bounds of the fitting parameters.

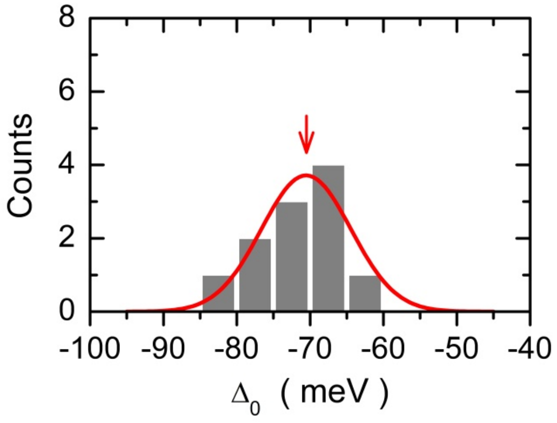

Supplementary Figure 5| Histogram of fitted Δ_0 for monolayer WSe_2. We analyzed the PL intensity-energy correlations from 11 $MoSe_2$-WSe_2 HJ flakes and obtain Δ_0 for unstrained WSe_2 from model fitting. By analyzing the distribution using a Gaussian function (red curve), we determine the average Δ_0 as -71 meV with a standard deviation of ~ 7 meV.

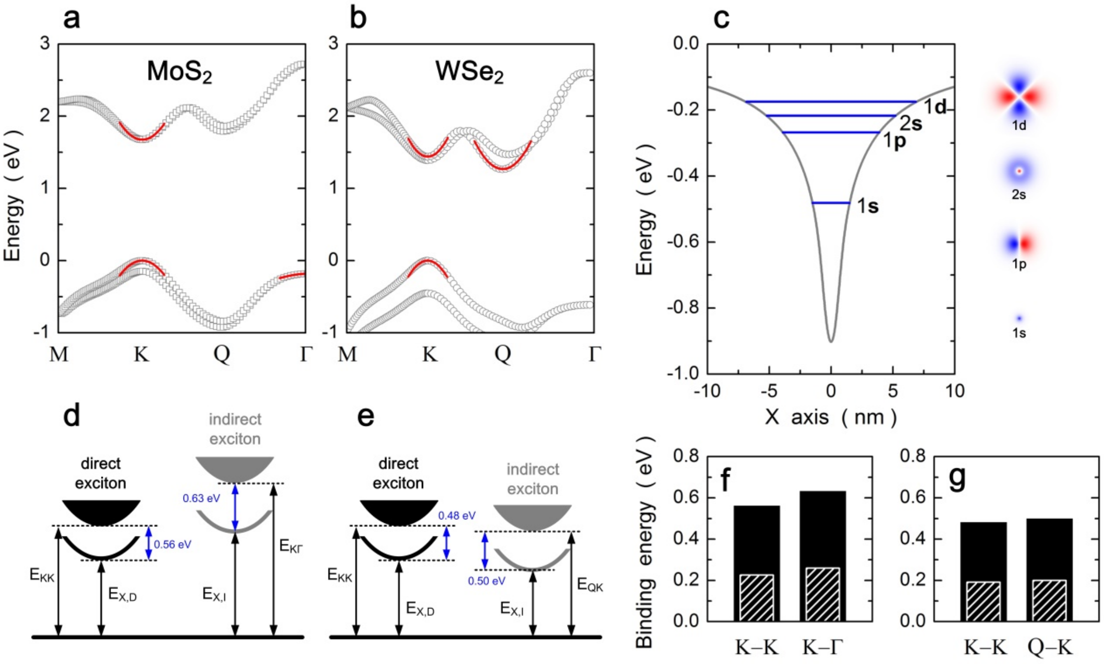

Supplementary Figure 6| Estimation of direct and indirect exciton binding energies. (a,b) Electronic band structures for monolayer MoS_2 (a) and WSe_2 (b) from DFT calculations. The electron and hole effective masses in different valleys are then determined by fitting the band curvatures with parabolic functions (red curves). (c) Effective Coulomb potential and calculated exciton states of a suspended WSe_2 monolayer, where the corresponding exciton wavefunctions are shown on the right panel. (d,e) Schematics of the direct and indirect exciton states for suspended monolayers MoS_2 (d) and WSe_2 (e), where the binding energies are labeled. (f,g) Effect of sapphire substrate on the exciton binding energy for monolayers MoS_2 (f) and WSe_2 (g), where the slash lines (black bars) are results with (without) sapphire substrates.

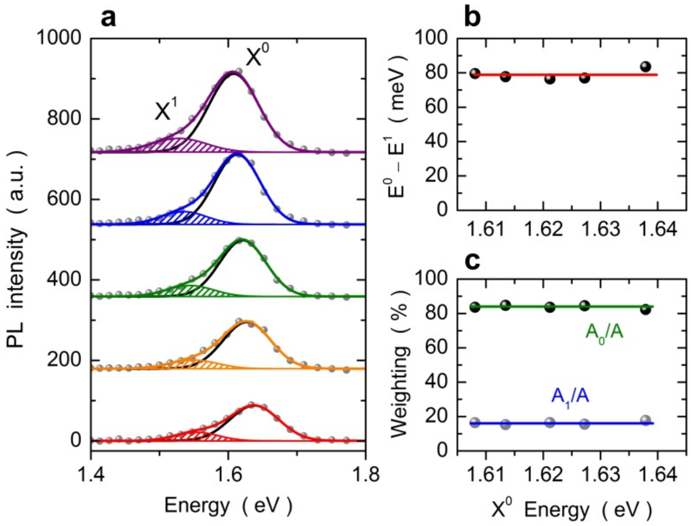

Supplementary Figure 7| Line shape analysis of PL from strained $MoSe_2$-WSe_2 HJs. (a) Analysis of the selected PL spectra of monolayer WSe_2 shown in Fig. 2e by double-Gaussian fits. (b) The energy difference and (c) relative weighting of X^0 and X^1 peaks as a function of X^0 energy.

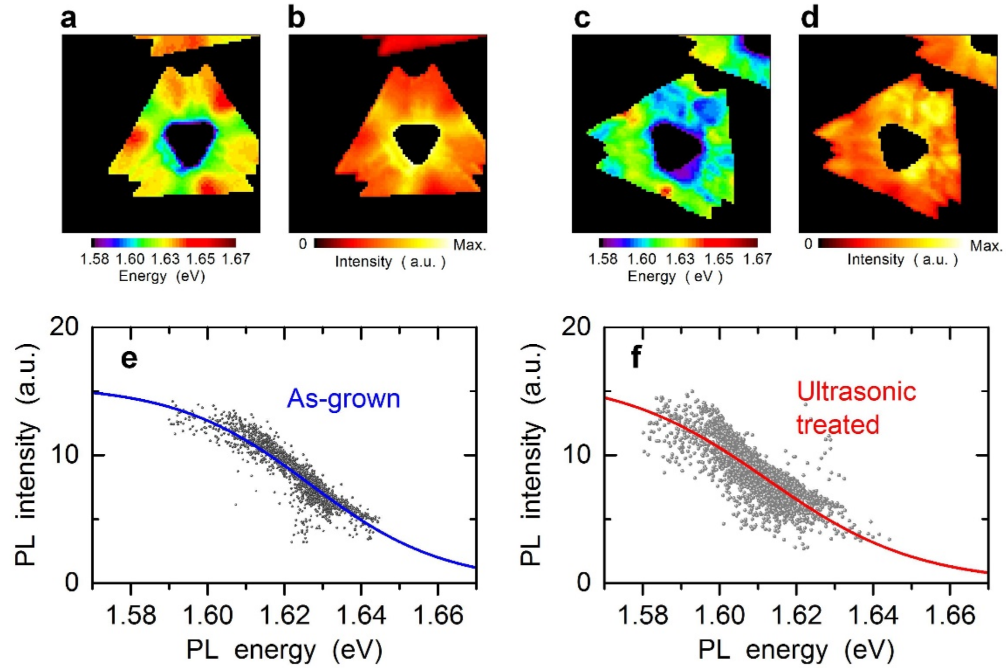

Supplementary Figure 8| PL inhomogeneity in $MoSe_2$-WSe_2 HJs before and after ultrasonic treatments. Contour color maps of PL energy (a,c) and intensity (b,d) in the WSe_2 region of a $MoSe_2$-WSe_2 lateral HJ measured before (a,b) and after (c,d) ultrasonic treatments. (e-f) The corresponding PL intensity-energy correlations and fittings for as-grown (e) and ultrasonic treated (f) samples.

Supplementary Note 1: Rate-equation model

The populations of direct ($N_{\mathrm{X,D}}$) and indirect ($N_{\mathrm{X,I}}$) excitons can be described by a simple rate-equation model, as shown in **Supplementary Fig. 1**. The rate equations for $N_{\mathrm{X,D}}$ and $N_{\mathrm{X,I}}$ can be expressed as

$$\frac{\mathrm{d}}{\mathrm{d}t}N_{\mathrm{X,D}} = g - \frac{N_{\mathrm{X,D}}}{\tau_{\mathrm{D}}} - \frac{N_{\mathrm{X,D}}}{\tau_{\mathrm{s}}}e^{-\frac{\Delta}{k_{\mathrm{B}}T}} + \frac{N_{\mathrm{X,I}}}{\tau_{\mathrm{s}}}, \qquad (1)$$

$$\frac{\mathrm{d}}{\mathrm{d}t}N_{\mathrm{X,I}} = -\frac{N_{\mathrm{X,I}}}{\tau_{\mathrm{I}}} + \frac{N_{\mathrm{X,D}}}{\tau_{\mathrm{s}}}e^{-\frac{\Delta}{k_{\mathrm{B}}T}} - \frac{N_{\mathrm{X,I}}}{\tau_{\mathrm{s}}}. \qquad (2)$$

where, g is the exciton generation rate, τ_{D} (τ_{I}) is the direct (indirect) exciton lifetime and the τ_{s} is the exciton intervalley scattering time, where $\tau_{\mathrm{D}}^{-1} = \tau_{\mathrm{Dr}}^{-1} + \tau_{\mathrm{nr}}^{-1}$ ($\tau_{\mathrm{I}}^{-1} = \tau_{\mathrm{Ir}}^{-1} + \tau_{\mathrm{nr}}^{-1}$) includes both the radiative lifetime τ_{Dr} (τ_{Ir}) and non-radiative lifetime τ_{nr} for the direct (indirect) excitons. In steady-state conditions, i.e., $\frac{\mathrm{d}}{\mathrm{d}t}N_{\mathrm{X,D}} = 0$ and $\frac{\mathrm{d}}{\mathrm{d}t}N_{\mathrm{X,I}} = 0$, we have

$$\frac{N_{\mathrm{X,D}}}{\tau_{\mathrm{D}}} + \frac{N_{\mathrm{X,D}}}{\tau_{\mathrm{s}}}e^{-\frac{\Delta}{k_{\mathrm{B}}T}} - \frac{N_{\mathrm{X,I}}}{\tau_{\mathrm{s}}} = g, \qquad (3)$$

$$\frac{N_{\mathrm{X,I}}}{\tau_{\mathrm{I}}} - \frac{N_{\mathrm{X,D}}}{\tau_{\mathrm{s}}}e^{-\frac{\Delta}{k_{\mathrm{B}}T}} + \frac{N_{\mathrm{X,I}}}{\tau_{\mathrm{s}}} = 0. \qquad (4)$$

From Eq. (4), we obtain

$$N_{\mathrm{X,I}}\left(1 + \frac{\tau_{\mathrm{s}}}{\tau_{\mathrm{I}}}\right) = N_{\mathrm{X,D}}\exp(-\Delta/k_{\mathrm{B}}T)\ . \qquad (5)$$

Since the excton intervalley scattering time τ_{s} is very short (subpicosecond range) compared with τ_{I} (nanosecond range) [5], the relationship between $N_{\mathrm{X,D}}$ and $N_{\mathrm{X,I}}$ can be reduced to the Boltzmann distribution:

$$N_{\mathrm{X,I}} \cong N_{\mathrm{X,D}}\exp\left(-\frac{\Delta}{k_{\mathrm{B}}T}\right). \qquad (6)$$

Using $N_{\mathrm{X,D}} + N_{\mathrm{X,I}} = N_0$ and $N_{\mathrm{X,I}} = N_{\mathrm{X,D}}\exp(-\Delta/k_{\mathrm{B}}T)$, we obtain

$$N_{\mathrm{X,D}} = \frac{N_0}{1 + \exp(-\Delta/k_{\mathrm{B}}T)}\,, \qquad (7)$$

$$N_{\mathrm{X,I}} = \frac{N_0}{1 + \exp(+\Delta/k_{\mathrm{B}}T)}\,. \qquad (8)$$

The direct-exciton PL intensity I_{PL} is given by

$$I_{\mathrm{PL}} \propto \frac{N_{\mathrm{X,D}}}{\tau_{\mathrm{Dr}}}\,. \qquad (9)$$

At room temperature, the exciton recombination is governed by the nonradiative recombination, i.e., $\tau_{\mathrm{Ir}} \gg \tau_{\mathrm{Dr}} \gg \tau_{\mathrm{nr}}$ and $\tau_{\mathrm{D}} \cong \tau_{\mathrm{I}} \cong \tau_{\mathrm{nr}}$. Eqs. (3) and (4) thus give $g\tau_{\mathrm{nr}} \cong N_{\mathrm{X,D}} + N_{\mathrm{X,I}} = N_0$. The PL intensity can be expressed as

$$I_{\mathrm{PL}} \propto \frac{N_{\mathrm{X,D}}}{\tau_{\mathrm{Dr}}} \cong \frac{g\left(\frac{\tau_{\mathrm{nr}}}{\tau_{\mathrm{Dr}}}\right)}{1 + \exp\left(-\frac{\Delta}{k_{\mathrm{B}}T}\right)}\,. \qquad (10)$$

If the strain-induced variation in $N_{\mathrm{X,D}}$ is more significant than the local variation in the ratio of $\tau_{\mathrm{nr}}/\tau_{\mathrm{Dr}}$, the measured I_{PL} can be correlated to the local variation in $N_{\mathrm{X,D}}$. According to the range of PL shifts in our samples and recent theoretical calculations [6], we estimated that the strain-induced changes in τ_{Dr} is small ($< 5\%$). While we don't have model to predict the spatial variation in the ratio of $\tau_{\mathrm{nr}}/\tau_{\mathrm{Dr}}$, it is reasonable to assume that $\tau_{\mathrm{nr}}/\tau_{\mathrm{Dr}}$ exhibits a normal distribution fluctuating around a certain value. From our experiments, the measured I_{PL} follows the function $\frac{N_0}{1+\exp(-\Delta/k_{\mathrm{B}}T)}$ very well, but with residuals exhibiting a normal distribution, as shown in Supplementary **Fig. S2**. The normal distributed residuals thus reflect the spatial variation in $\tau_{\mathrm{nr}}/\tau_{\mathrm{Dr}}$, giving rise to a fluctuation in I_{PL} of about $\pm 5\%$ (standard deviation), which is indeed less significant than the strain-induced variation in $N_{\mathrm{X,D}}$.

Supplementary Note 2: Estimation of direct and indirect exciton binding energies

Monolayer TMDs are known to exhibit very strong Coulomb interactions due to its two-dimensional nature and the reduced dielectric screening [7,8], giving rise to tightly bound excitons with anomalously large binding energies. We calculate the exciton binding energy based on the effective mass approximation with the effective 2D in-plane Coulomb potential $V_{2\mathrm{D}}(\rho)$ according to the Keldysh model [9,10,11],

$$V_{2\mathrm{D}}(\rho) = \frac{\pi e^2}{(\epsilon_1 + \epsilon_2)}\left[H_0\left(\frac{\rho}{\rho_0}\right) - Y_0\left(\frac{\rho}{\rho_0}\right)\right], \qquad (11)$$

where $\rho = (x^2 + y^2)^{1/2}$ is the in-plane charge separation, H_0 and Y_0 are the Struve function and the Bessel function of the second kind, $\epsilon_{1,2}$ are the dielectric constant of the top and bottom media, $\rho_0 = 2\pi\chi_{2\mathrm{D}}$ is the screening length, where $\chi_{2\mathrm{D}}$ is the 2D polarizability of the planar material. First, we determine the electron and hole effective masses by fitting the band curvatures with parabolic functions (**Figs. S5a-b**). Then, the exciton binding energy was calculated by solving the effective 2D potential using the finite-element method (**Fig. S5c**) [10]. In the calculation, we have modeled the dielectric TMD monolayers of MoS_2 ($\chi_{2\mathrm{D}} = 0.660$ nm) and WSe_2 ($\chi_{2\mathrm{D}} = 0.718$ nm) sandwiched between the top vacuum ($\epsilon = 1$) and bottom sapphire substrate ($\epsilon = 3.1$) [10,12], and the effect of sapphire substrate on the binding energy is then discussed.

We start the discussion for the case of suspended TMD monolayer in vacuum. For monolayer MoS_2 (**Fig. S5d**), we obtained binding energies of $E_{\mathrm{B,D}} = 0.56$ eV (direct K-K) and $E_{\mathrm{B,I}} = 0.63$ eV (indirect K-Γ), where the larger K-Γ binding energy originates from the much massive hole at Γ-valley. For monolayer WSe_2 (**Fig. S5e**), we obtained binding energies of $E_{\mathrm{B,D}} = 0.48$ eV (direct K-K) and $E_{\mathrm{B,I}} = 0.50$ eV (indirect Q-K), where the similar binding energies are caused by the similar effective electron masses at K- and Q-valleys. For the sapphire-supported TMDs, the

exciton binding energy is significantly reduced due to the increased dielectric screening [11]. As shown in **Figs. S5f-g**, we obtained $E_{\mathrm{B,D}} = 0.23$ eV (direct K-K) and $E_{\mathrm{B,I}} = 0.26$ eV (indirect K-Γ) for monolayer MoS_2, and $E_{\mathrm{B,D}} = 0.19$ eV (direct K-K) and $E_{\mathrm{B,I}} = 0.20$ eV (indirect Q-K) for monolayer WSe_2. It's noting that although the exciton binding energies are significantly reduced by >300 meV, the resulting differences between $E_{\mathrm{B,D}}$ and $E_{\mathrm{B,I}}$ are not changed too much.

Supplementary References:

1. Amani, M. *et al.* Growth-substrate induced performance degradation in chemically synthesized monolayer MoS_2 field effect transistors, *Appl. Phys. Lett.* **104**, 203506 (2014).
2. Liu, Z. *et al.* Strain and structure heterogeneity in MoS_2 atomic layers grown by chemical vapour deposition, *Nat. Commun.* **5**, 5246 (2014).
3. Park, K.-D. *et al.* Hybrid tip-enhanced nanospectroscopy and nanoimaging of monolayer WSe_2 with local strain control, *Nano Lett.* **16**, 2621-2627 (2016).
4. Amani, M. *et al.* High luminescence efficiency in MoS_2 grown by chemical vapor deposition, *ACS Nano* **10**, 6535-6541 (2016).
5. Selig, M. *et al.* Dark and bright exciton formation, thermalization, and photoluminescence in monolayer transition metal dichalcogenides, Preprint at http://arxiv.org/abs/1703.03317v1 (2017).
6. Feierabend, M., Morlet, A., Berghäuser, G. & Malic, E. Impact of strain on the optical fingerprint of monolayer transition metal dichalcogenides, Preprint at http://arxiv.org/abs/1706.00491v1 (2017).
7. Zhang, C., Johnson, A., Hsu, C.-L., Li, L.-J. & Shih, C.-K. Direct imaging of band profile in single layer MoS_2 on graphite: Quasiparticle energy gap, metallic edge states, and edge band bending, *Nano Lett.* **14**, 2443-2447 (2014).
8. He, K. *et al.* Tightly bound excitons in monolayer WSe_2, *Phys. Rev. Lett.* **113**, 026803 (2014).
9. Keldysh, L. V. Coulomb interaction in thin semiconductor and semimetal films, *JETP Lett.* **29**, 658-660 (1979).
10. Berkelbach, T. C., Hybertsen, M. S. & Reichman, D. R. Theory of neutral and charged excitons in monolayer transition metal dichalcogenides, *Phys. Rev. B* **88**, 045318 (2013).
11. Zhu, X. *et al.* Charge transfer excitons at van der Waals interfaces, *J. Am. Chem. Soc.* **137**, 8313-8320 (2015).
12. DeFranzo, A. C. & Pazol, B. G. Index of refraction measurement on sapphire at low temperatures and visible wavelengths. *Appl. Opt.* **32**, 2224-2234 (1993).
